# Long-Term Evidence of ENSO-Driven Rodent Population Dynamics in a Natural Plague Focus of Southwestern China

**DOI:** 10.3390/ani16152388

**Published:** 2026-08-03

**Authors:** Chao Su, Yongman Guo, Yunqin Shen, Yuqiong Li, Liqiong Su, Lei Xu, Zihou Gao

**Affiliations:** 1 Yunnan Institute of Endemic Diseases Control and Prevention, Kunming 650500, China; 2 Yunnan Provincial Key Laboratory of Natural Foci Disease Prevention and Control, Kunming 650500, China; 3 Faculty of Engineering, Henan University, Zhengzhou 450046, China; 4 Vanke School of Public Health, Tsinghua University, Beijing 100084, China

**Keywords:** El Niño–Southern Oscillation (ENSO), rodent population dynamics, flea–host interactions, natural plague focus

## Abstract

Climate variability can modulate zoonotic disease risk by altering interactions among wildlife hosts, vectors, and human environments, yet long-term evidence linking large-scale climate oscillations to natural plague systems remains scarce. We investigated the influence of El Niño–Southern Oscillation (ENSO)-related climate variability on rodent host dynamics in a long-established plague focus in Jianchuan County, southwestern China. Using continuous monthly surveillance data from 1978 to 2025, we analyzed population dynamics of two ecologically distinct plague hosts—the wild rodent *Apodemus chevrieri* and the domestic rodent *Rattus tanezumi*—together with flea infection rates and local climate variables. Generalized additive models showed that ENSO, quantified by the Southern Oscillation Index (SOI), exerted significant delayed effects on both rodent populations, with El Niño conditions consistently associated with increased host abundance. Wavelet coherence analyses revealed synchronized ENSO–rodent oscillations at dominant 2–3-year periodicities, indicating persistent large-scale climate forcing. In contrast, responses to local environmental factors differed between species: surface temperature strongly constrained the wild rodent *A. chevrieri*, whereas the domestic *R. tanezumi* showed weaker thermal sensitivity, consistent with buffering by human-modified indoor habitats. Flea infection rates declined as rodent densities increased, suggesting a dilution effect within the host–vector system. By integrating long-term wildlife surveillance, climate indicators, and vector data, this study provides empirical evidence that ENSO-driven climate variability plays a central role in regulating plague source activity at the human–animal–environment interface. These findings highlight the value of climate-informed, ecology-based surveillance frameworks for anticipating periods of elevated plague risk and strengthening early warning systems in endemic regions.

## Simple Summary

Plague remains a serious disease in some parts of the world because it is maintained in wild rodents and transmitted by fleas. Changes in climate can alter the abundance of these animals and therefore influence the risk of disease transmission. However, long-term evidence of how large-scale climate variability affects plague host populations is still limited. In this study, we analyzed 46 years of continuous rodent surveillance data collected from Jianchuan County, a major natural plague focus in southwestern China. We investigated how changes in regional climate influenced the population dynamics of two important rodent species that carry the plague bacterium. We found that periods associated with warmer and wetter climate conditions were generally followed by increases in rodent populations, although the two species responded differently to local environmental conditions because they occupy different habitats. We also found that fluctuations in rodent populations followed regular climate cycles over time. These findings improve our understanding of how climate influences wildlife hosts of plague and demonstrate that long-term climate information can help predict periods when rodent populations are more likely to increase. This knowledge may contribute to earlier disease surveillance, more effective wildlife monitoring, and improved public health preparedness.

## 1. Introduction

Climate change has profoundly altered terrestrial ecosystems through increased frequencies of extreme weather events [1], biodiversity loss [2], population fluctuations [3,4], and heightened risks of zoonotic disease emergence and spillover [5]. Among the major drivers of interannual climate variability, the El Niño–Southern Oscillation (ENSO) plays a central role in regulating interannual variability in global climate systems. ENSO is commonly quantified using the Southern Oscillation Index (SOI), which is derived from atmospheric pressure differences between Tahiti and Darwin, Australia, and is widely recognized as a key indicator of large-scale ocean–atmosphere interactions [6]. ENSO events reorganize atmospheric circulation patterns, producing widespread anomalies in precipitation and temperature that influence ecosystem processes across continents [7]. Although ENSO does not determine local climatic conditions directly, it substantially increases the probability of departures from long-term climatic averages [8], thereby influencing the dynamics of wildlife populations and the transmission of climate-sensitive infectious diseases.

Rodent populations exhibit a variety of life history strategies across environmental gradients, making them particularly sensitive to climate variability [9]. Rather than acting directly on rodent populations, ENSO primarily affects rodent demography through bottom-up ecological processes [10]. Changes in precipitation and temperature regulate primary productivity and vegetation growth, thereby altering food availability, reproductive success, juvenile survival, and ultimately population abundance [11]. Consequently, ENSO-driven rodent population dynamics have been documented in a variety of ecosystems worldwide. In western South America, El Niño events trigger rapid population increases in small rodents, whereas larger species respond after a longer delay [12]. Similar climate-driven fluctuations have been reported in semiarid grasslands of Inner Mongolia, where ENSO-associated changes in vegetation synchronized the population dynamics of sympatric rodent species [13], and in the Dongting Lake region of China, where ENSO-driven precipitation promoted outbreaks of the Yangtze vole (*Microtus fortis calamorum*) [4]. Comparable climate–rodent associations have also been documented in East and southern Africa, where rainfall variability regulates the breeding activity and population dynamics of the Natal multimammate mouse (*Mastomys natalensis*) in East and southern Africa, an important reservoir host of plague and several other zoonotic pathogens [14]. 

Rodent population dynamics are tightly linked to zoonotic disease risk, particularly plague, whose transmission depends on complex interactions among rodent hosts, flea vectors, environmental conditions, and humans [15]. Previous studies have shown that plague risk generally increases during periods of high rodent abundance because expanding host populations facilitate the maintenance and spread of *Yersinia pestis* within natural reservoirs [16]. Jianchuan County, Yunnan Province, represents one of the most important natural plague foci in southwestern China [17]. Following the first detection of *Y. pestis* antibodies in sheepdog serum in 1974, long-term surveillance confirmed two ecologically distinct plague systems: a domestic plague focus maintained by *Rattus tanezumi* around human settlements and a wild plague focus maintained by *Apodemus chevrieri* in farmlands and forest ecosystems [18]. The two rodent species are the dominant plague reservoir hosts in this region. Their major flea species, *Neopsylla specialis* (associated with *A. chevrieri*) and *Xenopsylla cheopis* (associated with *R. tanezumi*), are both well-recognized competent vectors of *Y. pestis* and therefore represent an important transmission bridge between wildlife and humans [18]. Human plague cases in Yunnan are typically initiated following epizootics in rodent populations, during which infected fleas seek alternative hosts after rodent mortality. Consequently, understanding how climate variability influences rodent host populations is fundamental for predicting subsequent changes in plague transmission risk. 

Although ENSO effects on rodent population dynamics have been studied in several ecosystems, three important knowledge gaps remain. First, most previous studies have relied on relatively short-term observations, limiting our understanding of how repeated ENSO cycles shape long-term rodent population dynamics [19]. Second, while climate, rodents, and plague vectors have often been investigated separately, few studies have simultaneously integrated long-term rodent surveillance, flea infection dynamics, and large-scale climate variability within a natural plague focus, despite these components representing the key ecological processes underlying plague persistence. Third, the mechanisms responsible for species-specific responses of sympatric rodent hosts to ENSO-driven climatic variability remain poorly understood. Addressing these gaps is essential for understanding how climate variability influences plague ecology and for developing climate-informed surveillance strategies under ongoing global climate change.

In this study, we investigated 47 years (1978–2025) of continuous rodent surveillance, flea infection records, and large-scale climate data to investigate how ENSO-driven climate variability regulates the population dynamics of two sympatric rodent species, *A. chevrieri* and *R. tanezumi*, in Jianchuan County. Specifically, we evaluated the relative and lagged effects of SOI, precipitation, temperature, and flea infection rates using generalized additive models and time-frequency analyses. We aimed to test: (1) whether ENSO acts as a dominant large-scale climate driver of rodent population fluctuations; (2) whether climate effects differ systematically between wild and domestic species with contrasting life histories. By elucidating these mechanisms, our study provides insights into how climate variability shapes plague source dynamics in southwestern China and informs early-warning strategies for rodent-borne disease risk under ongoing climate change.

## 2. Materials and Methods

### 2.1. Study Area

Jianchuan County, located in the central Hengduan Mountains of southwestern China (26°12′–26°41′ N, 99°33′–100°33′ E), represents one of the most active natural plague foci in southern China, and there are also intermittent outbreaks of epidemic situations among animals [18]. The region is situated at elevations ranging from approximately 2100 to 3000 m above sea level and characterized by a mosaic of agricultural landscapes, fragmented shrublands, secondary pine forests, and rural settlements [20]. Cultivated land is dominated by potato, maize and barley fields, interspersed with natural grasslands and shrub vegetation, providing suitable habitats for both wild and commensal rodent species. According to long-term meteorological records, the region has a mean annual temperature of approximately 12.3 °C and mean annual precipitation of approximately 724 mm, with nearly 80% of rainfall occurring during the monsoon season (May–October) [21]. These heterogeneous habitats support stable populations of plague reservoir hosts and their flea vectors.

### 2.2. Rodent Surveillance 

Rodent surveillance was conducted following standardized protocols from the World Health Organization (WHO) Plague Manual [22] and the Chinese national plague surveillance protocol [23]. The trapping equipment, sampling frequency, trapping locations, and rodent identification procedures remained unchanged throughout the study period under the standardized plague surveillance program, ensuring long-term comparability of the data. All surveillance personnel received standardized training according to the national plague surveillance guidelines. Species identification was performed by experienced experts based on morphological characteristics using standard taxonomic keys [24]. Data were independently checked before entry into the surveillance database, and annual quality-control audits were conducted by the Chinese Disease Control and Prevention to ensure consistency across years. Fried cereal grains and peanuts were used as bait. Traps were placed at intervals of approximately 5–10 m depending on habitat structure and were deployed before sunset and retrieved the following morning (approximately 12 h trapping duration). At least 300 traps were set each month across representative indoor and outdoor habitats, resulting in a minimum trapping effort of approximately 3600 trap-nights per year. Live trapping was performed monthly from January 1978 to December 2025. Rodent abundance was quantified using capture rates (number of rodents captured per trap).

We focused on the two dominant host species in the system: *Apodemus chevrieri*, the principal outdoor species inhabiting cultivated land and shrub ecosystems, and *Rattus tanezumi*, the dominant indoor commensal species. Rodent population density was defined as the number of rodents captured per trap using R = Nr/Ne × 100%, where Nr and Ne are the number of rodents captured and the number of effective traps, respectively. 

### 2.3. Flea Infection Rate and Flea Index

Immediately after rodent capture, ectoparasitic fleas were combed from each animal using fine forceps and flea combs. Fleas were preserved in 75% ethanol and identified to species under a stereomicroscope using standard taxonomic keys employed by the Chinese plague surveillance program. For each rodent species, flea infection rate was calculated as the proportion of captured rodents harboring fleas (rodents carrying fleas/total rodents), which refers to flea prevalence rather than flea abundance. Flea index was calculated as the mean number of fleas per captured rodent (total number of fleas collected/total rodents). The flea index represents the average ectoparasite burden experienced by the rodent population and is used as an indicator of vector abundance in plague surveillance.

### 2.4. Climate and ENSO Indices

ENSO variability was quantified using the Southern Oscillation Index (SOI), a standardized measure of air pressure differences between Tahiti and Darwin. We selected the SOI rather than the Niño 3.4 sea-surface temperature index because SOI directly reflects atmospheric circulation anomalies associated with ENSO and the atmospheric teleconnections represented by SOI are more closely linked to precipitation variability in southwestern China than sea-surface temperature anomalies alone. Moreover, SOI provides a continuous monthly index that facilitates lagged ecological analyses over long time periods. Sustained negative (positive) SOI values correspond to El Niño (La Niña) conditions [25]. Monthly SOI data were obtained from the National Centers for Environmental Information (Figure S1A). Regional temperature and precipitation data were derived from the NCEP–NCAR reanalysis dataset at 2.5° resolution (Figure S1B) and Climatic Research Unit Time Series (CRU TS v4) at 0.5° resolution (Figure S1C), respectively. We used reanalysis products rather than local meteorological observations because they provide spatially continuous and temporally homogeneous climate estimates over the entire study period (1978–2025), minimizing inconsistencies caused by station relocation, missing observations, or instrument replacement. Moreover, reanalysis datasets better represent climatic conditions across the heterogeneous mountainous landscape with varying altitudes than measurements from a single meteorological station. Climate variables were matched temporally to rodent and flea observations.

### 2.5. Statistical and Time-Frequency Analyses

Variance inflation factors (VIFs) were calculated prior to model fitting, and variables with VIF < 5 were considered free of problematic multicollinearity. We applied cross-correlation function (CCF) analysis to assess time-lagged relationships among ENSO (SOI), regional climate variables, flea infection rates, and rodent population densities [26]. Species-specific lag structures were detected, with dominant ENSO effects occurring within several months, consistent with rodent reproductive and demographic processes. Significant associations were detected between SOI and rodent capture rates, with dominant lags of approximately 7 months for *A. chevrieri* and 4 months for *R. tanezumi* (Figure S2). Months with missing surveillance observations were excluded from analyses. Climate variables contained no missing values.

Rodent population dynamics were modeled using generalized additive models (GAMs) with Poisson error distributions and log link functions, allowing for nonlinear climate effects [27]. As shown below: $log[E(Yt\;)]=A_{i}+f_{1}\;(SOIt-lags\;)+f_{2}\;(TEM\;)+f_{3}\;(PRE\;)+f_{4}\;(Flea\;)+f_{5}(Flea\;index)$, where *Yt* represents rodent density; *A_i_* is a row of the model matrix for any strictly parametric model components, and *f_i_* () are smooth functions of SOI (SOI), temperature (TEM), precipitation (PRE), flea infection rate (Flea) and flea index on rodent population dynamics. Variables that were not statistically significant were removed through backward model selection based on the Akaike Information Criterion (AIC) and generalized cross-validation (GCV), resulting in the final species-specific models. Model complexity was quantified using the estimated effective degrees of freedom (edf) for each smooth term. An edf close to 1 indicates an approximately linear relationship, whereas values greater than 1 indicate increasingly nonlinear responses. In the final models, edf values ranged from 1.000 to 1.968, suggesting that both linear and nonlinear climate–rodent relationships were present depending on the predictor variable (Table S1). In these models, rodent population density was specified as the response variable because the primary objective of this study was to evaluate how large-scale climate variability, local environmental conditions, and flea-related factors were associated with the dynamics of the major plague host populations. In addition, we conducted complementary GAM analyses in which flea infection rate was treated as the response variable and SOI, precipitation, surface temperature, and host population density were included as explanatory variables (Figures S3 and S4). These analyses showed that flea infection rates were not significantly associated with SOI or precipitation in either species. Therefore, flea-related variables were treated as potential predictors rather than response variables.

To further explore nonstationary relationships between ENSO and rodent dynamics, cross-wavelet and wavelet coherence analyses were conducted using the WaveletComp package (version 1.2) [28,29]. Because all datasets consisted of monthly observations, while periodicities were expressed in years, the temporal sampling interval (dt) was set to 1/12, corresponding to one month. The scale resolution parameter (dj) was set to 1/250, following the package recommendations for high-resolution analyses of long-term climate time series, thereby allowing fine discrimination of periodic signals while maintaining computational stability. Monthly rodent abundance series (1978–2025) and monthly SOI data (1977–2025) were analyzed to identify temporal periodicities and time-varying coherence between ENSO variability and rodent population dynamics. Statistical significance was assessed against red-noise backgrounds using Monte Carlo simulations (*p* < 0.05). Arrows indicate phase relationships, with rightward arrows denoting in-phase and leftward arrows denoting anti-phase dynamics. All analyses were performed in the R environment (Version 4.4.1) with statistical significance set at *p* < 0.05 [30]. 

## 3. Results

### 3.1. Long-Term Trends in Rodent Population Dynamics

**Figure 1:**
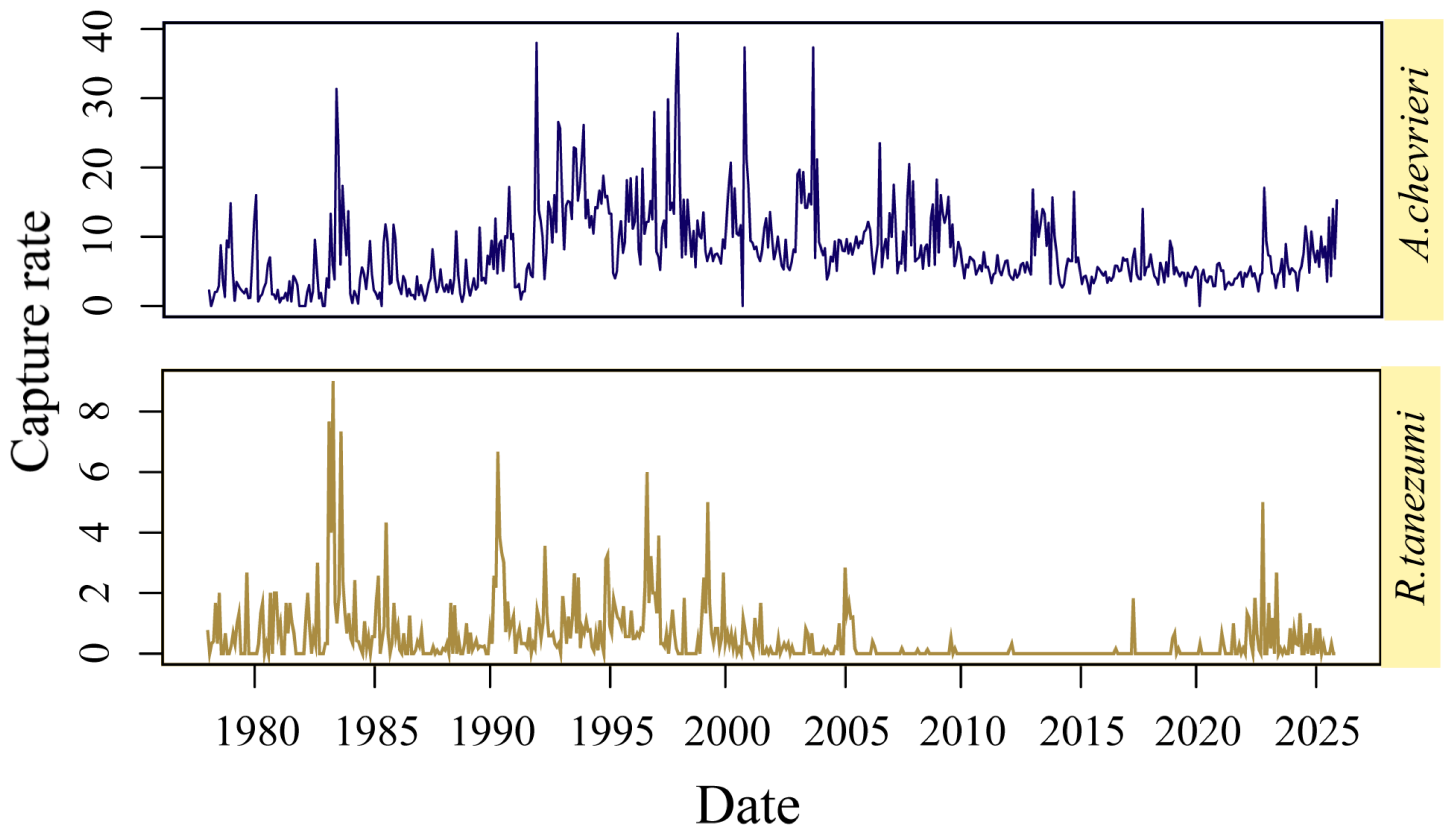
Monthly capture rates of *A. chevrieri* and *R. tanezumi* from 1978 to 2025.

Before examining the effects of climate variables, we first characterized the long-term population dynamics of the two dominant plague-host species (Figure 1). Both *A. chevrieri* and *R. tanezumi* exhibited pronounced interannual fluctuations throughout the surveillance period (1978–2025), although their temporal patterns differed. The population density of *A. chevrieri* showed no significant monotonic temporal trend over the full surveillance period, despite recurrent multi-year fluctuations and episodic increases in abundance. In contrast, *R. tanezumi* exhibited a significant temporal decline, particularly after approximately 2005, followed by a partial recovery during the later surveillance period. Formal trend and time-series analyses therefore indicated that the two host species differed not only in the magnitude of their temporal variability but also in their long-term population trajectories. These contrasting dynamics provided the basis for subsequently examining whether large-scale climate variability and local ecological factors contributed to species-specific population changes.

### 3.2. Climate and Vector Effects on Rodent Population Dynamics

Our analysis revealed that rodent population dynamics in Jianchuan were significantly associated with both large-scale climate variability and local environmental and vector-related factors. The results revealed marked species-specific differences in both the strength and significance of these associations.

**Figure 2:**
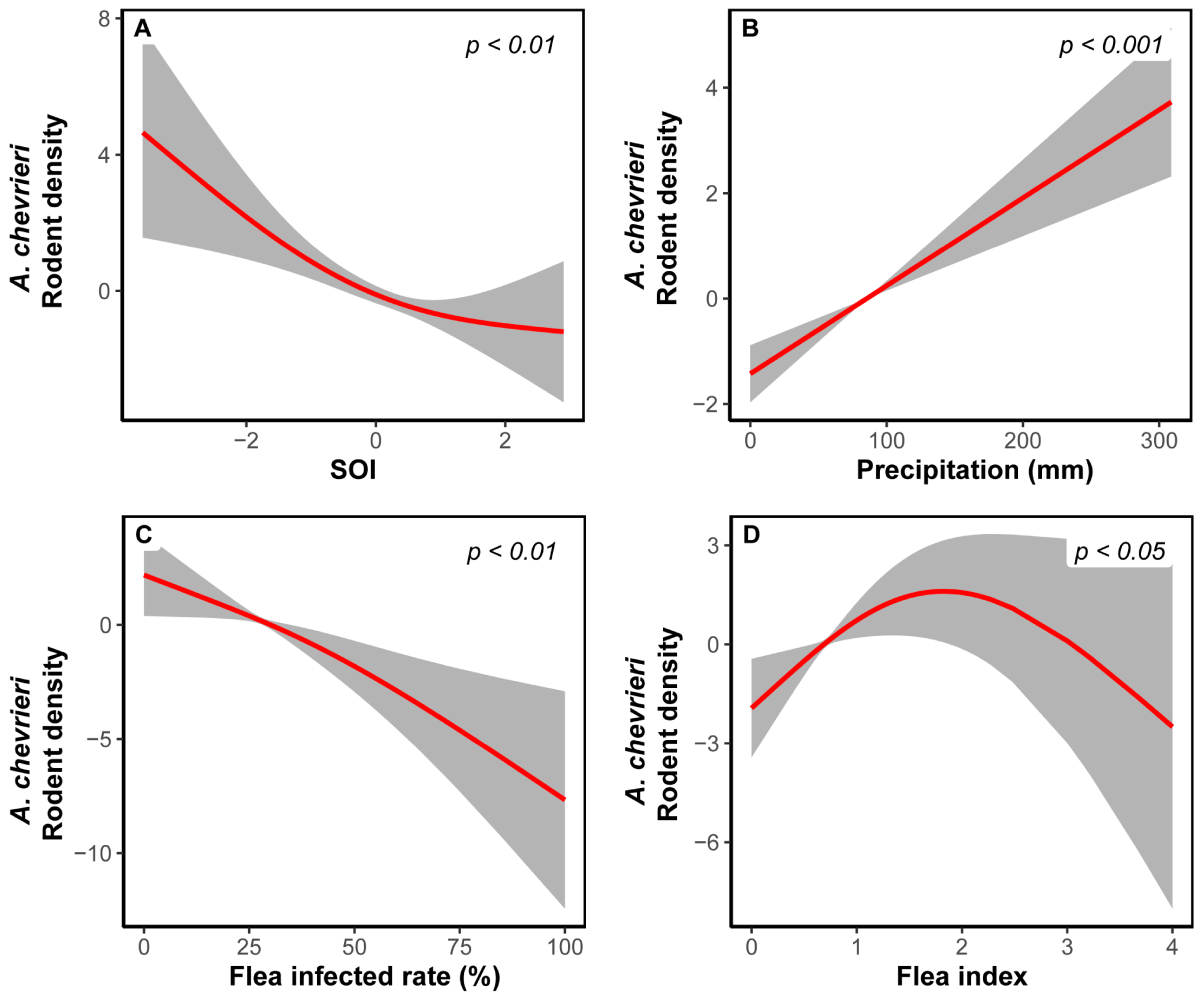
Best-fitting Generalized additive models describing the associations between rodent density in *A. chevrieri* and SOI (**A**), precipitation (**B**), flea infected rate (**C**), and flea index (**D**). Red segments indicate statistically significant effects (*p* < 0.05); shaded areas denote 95% confidence intervals. SOI < 0 indicates an El Niño episode; SOI > 0 indicates a La Niña event.

For *A. chevrieri*, population density was strongly influenced by both climate and flea-related variables (Figure 2). The SOI exhibited a significant negative relationship with population density (edf = 1.63, *p* < 0.01; Figure 2A), indicating that El Niño conditions (negative SOI values) were associated with increased rodent abundance. Precipitation showed a strong positive linear association with rodent density (edf = 1.00, *p* < 0.001; Figure 2B), suggesting that rodent density increased steadily across the rainfall gradient.

Vector-related variables also played a significant role. Flea infection rate was negatively associated with population density (edf = 1.41, *p* < 0.01; Figure 2C), consistent with a dilution effect as host populations increased. In contrast, the flea index showed a nonlinear (hump-shaped) relationship with *A. chevrieri* abundance (*p* < 0.05; Figure 2D), with peak rodent density occurring at intermediate flea burdens (approximately index 2), indicating that moderate flea burdens were associated with the highest host population densities, whereas heavier infestations corresponded to reduced rodent abundance.

**Figure 3:**
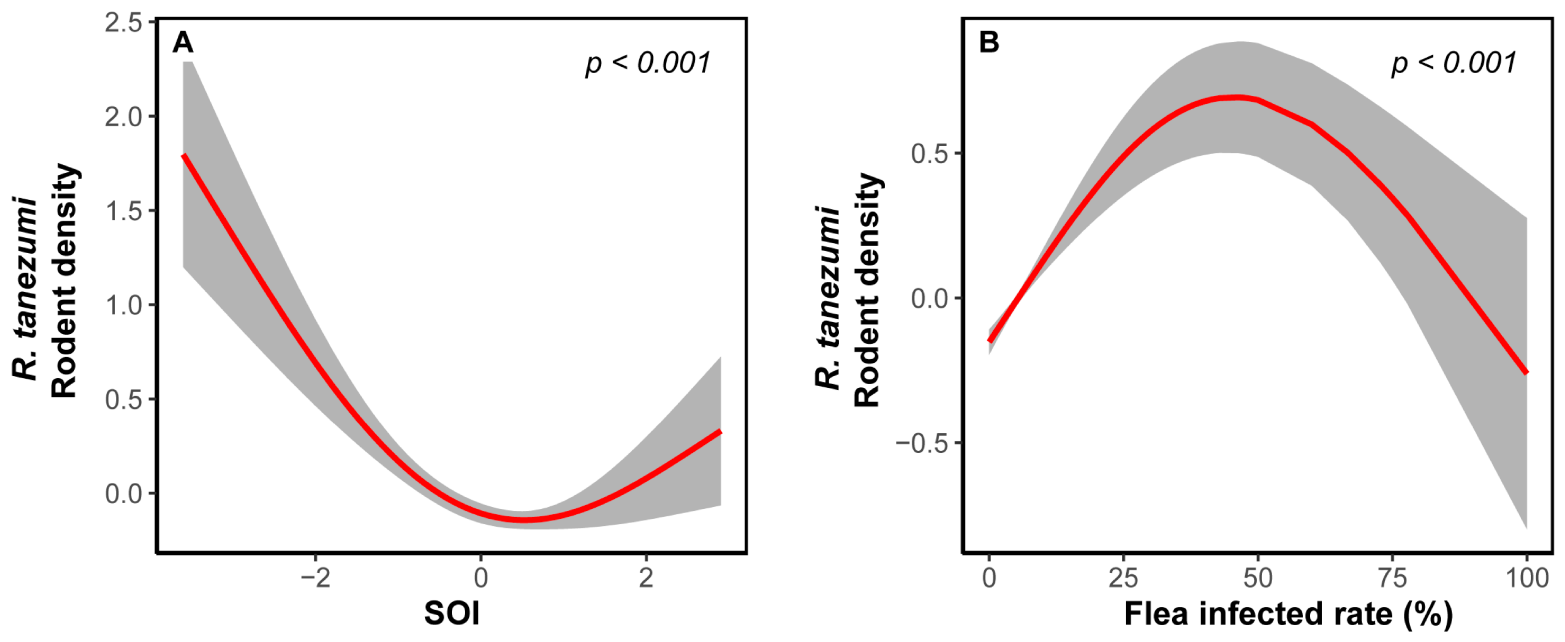
Best-fitting Generalized additive models describing the associations between rodent density in *R. tanezumi* and SOI (**A**), and flea infected rate (**B**). Red segments indicate statistically significant effects (*p* < 0.05); shaded areas denote 95% confidence intervals.

For *R. tanezumi*, population dynamics were significantly associated with both large-scale climate variability and vector-related factors (Figure 3). SOI exhibited a nonlinear relationship with *R. tanezumi* abundance (edf = 1.95, *p* < 0.001; Figure 3A), with rodent density lowest at intermediate SOI values and higher under both El Niño (negative SOI) and La Niña (positive SOI) extremes. Flea infection rate was also significantly associated with *R. tanezumi* abundance (edf = 1.97, *p* < 0.001; Figure 3B). The fitted smooth function showed a hump-shaped relationship: rodent population density increased as flea infection rate increased from low levels, reaching a maximum at approximately 50% flea prevalence, after which population density declined sharply with further increases in flea infection rate. This nonlinear pattern suggests that moderate flea infestation coincided with relatively high rodent abundance, whereas high flea infection rates were associated with reduced host population density.

### 3.3. Temporal Dynamics and ENSO-Driven Periodicity

**Figure 4:**
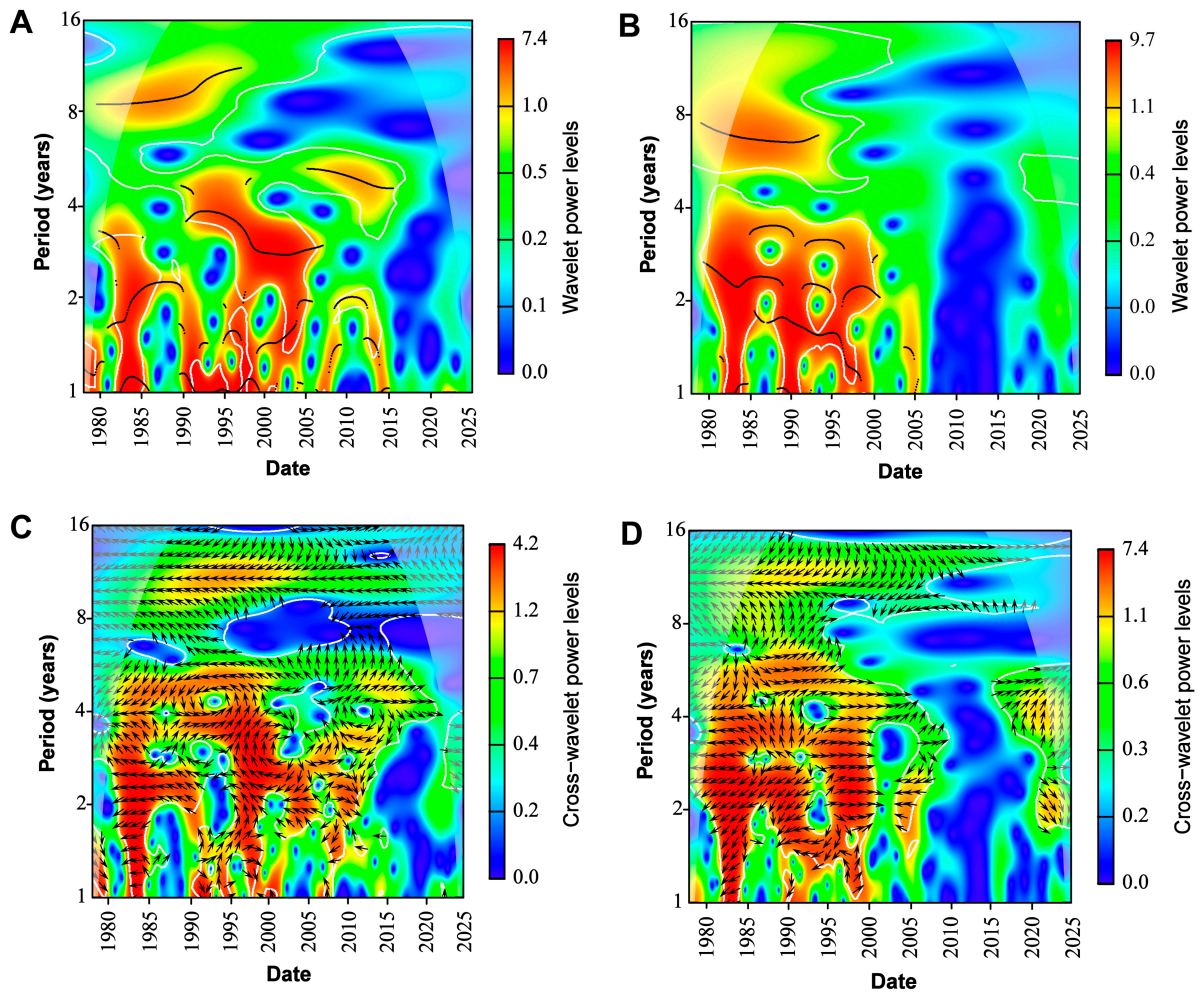
Continuous wavelet power spectra for *A. chevrieri* (**A**) and *R. tanezumi* (**B**) from 1978 to 2025, and wavelet coherence between SOI and the population dynamics of *A. chevrieri* (**C**) and *R. tanezumi* (**D**). Color intensity represents wavelet power (**A**,**B**) or cross-wavelet power (**C**,**D**), with red color indicating stronger oscillatory signals. White contours indicate regions exceeding the 95% confidence level based on red-noise background spectra. Black curves indicate the cone of influence, outside which edge effects may reduce reliability. The arrows indicate the relative phase relationship, and when the arrow points to the right, it indicates an in-phase relationship, otherwise, it indicates an anti-phase relationship.

Continuous wavelet analyses revealed pronounced temporal variability in both rodent population dynamics and large-scale climate forcing. The population dynamics of *A. chevrieri* exhibited strong and persistent oscillatory power primarily within the 2–4-year band throughout the study period, with intermittent signals extending to longer periodicities (6–8 years) (Figure 4A). Similarly, *R. tanezumi* displayed dominant periodicities concentrated in the 2–4-year range, particularly before the early 2000s, after which wavelet power declined substantially, consistent with the reduction in observed population density (Figure 4B).

Cross-wavelet coherence analyses demonstrated significant synchronization between rodent population dynamics and the ENSO variability, particularly within the 2–3-year periodic band (Figure 4C,D). High coherence regions (red and yellow areas within the 95% confidence contours) indicate that ENSO variability acts as a dominant large-scale driver of rodent fluctuations at these timescales. Prior to the mid-1990s, the population dynamics of both *A. chevrieri* and *R. tanezumi* were predominantly in anti-phase with SOI, indicating increased rodent abundance during El Niño conditions. Following this period, the phase structure became more heterogeneous, with intermittent in-phase relationships, suggesting a restructuring of climate–population coupling. Notably, coherent oscillations between SOI and *R. tanezumi* weakened markedly after 2005, coinciding with the sharp decline in its population density. After 2015, coherent oscillations between SOI and *R. tanezumi* began to intensify, which was related to the increase in the population density (Figure 4D).

## 4. Discussion

Using nearly five decades of continuous rodent surveillance from a well-characterized natural plague focus in southwestern China, we show that large-scale climate variability associated with the ENSO exerts a persistent and species-specific influence on rodent population dynamics. Across both *A. chevrieri* and *R. tanezumi*, population densities were consistently negatively associated with the SOI, indicating increased rodent abundance during El Niño conditions. Importantly, wavelet analyses revealed that the strength of climate–rodent coupling varied over time, indicating that anthropogenic disturbance may modify climatic regulation of plague host populations. These findings provide new evidence that long-term climate oscillations contribute to the ecological processes underlying plague maintenance and support the incorporation of climate information into One Health surveillance systems.

### 4.1. ENSO Synchronizes Rodent Population Dynamics Through Delayed Climatic Pathways

ENSO-related climate variability has been shown to modulate temperature and precipitation regimes across East Asia, particularly during winter and spring [31]. In China, El Niño events are often associated with warmer winters and increased precipitation, conditions that can enhance primary productivity and improve overwinter survival of small mammals [13]. Our findings are consistent with previous reports linking El Niño episodes to rodent outbreaks in South America and China and suggest that ENSO functions as a large-scale synchronizing force on rodent populations [3,8,13]. ENSO does not directly influence rodent populations. Instead, it modifies regional precipitation and temperature regimes, which subsequently affect vegetation productivity, food availability, reproductive success, juvenile survival, and ultimately population recruitment [13]. These ecological processes require several months before becoming detectable at the population level, providing a mechanistic explanation for the observed lagged relationship. Similar delayed responses between ENSO and rodent outbreaks have been reported for *Phyllotis darwini* in Chile [32], *Mastomys natalensis* in East Africa [14], and *Microtus fortis* in the Dongting Lake region of China [4], suggesting that delayed demographic responses may represent a general ecological feature of climate-driven rodent systems.

### 4.2. Species-Specific Responses Reveal Distinct Ecological Mechanisms of Plague Hosts

Despite shared sensitivity to ENSO, *A. chevrieri* and *R. tanezumi* exhibited markedly different responses to local environmental and vector-related factors, reflecting differences in habitat use and life-history strategies. For *A. chevrieri*, a wild species inhabiting farmlands and forest ecosystems, population dynamics was shaped by a combination of climate and vector-related variables [21]. The strong positive association with precipitation suggests that water availability enhances primary productivity and food resources, thereby supporting population growth. In addition, the significant relationships with flea infection rate and flea index indicate that host–vector interactions play a regulatory role in population dynamics. The observed decline in flea infection rate with increasing rodent density is consistent with a potential dilution pattern, while the nonlinear response to flea index suggests a threshold beyond which vector burden constrains host population growth [33].

*Rattus tanezumi*, a commensal species closely associated with human settlements, exhibited population dynamics that were primarily associated with large-scale climatic variability, particularly ENSO, while also showing a significant nonlinear relationship with flea infection rate. Unlike the wild species *A. chevrieri*, *R. tanezumi* inhabits human dwellings and surrounding peridomestic environments, where relatively stable indoor microclimates and continuous food resources buffer the direct effects of local environmental fluctuations. Consequently, broad-scale climatic oscillations such as ENSO are more likely to influence its population indirectly through changes in agricultural production, human activities, and resource availability rather than through immediate changes in local habitat conditions. The nonlinear association between flea infection rate and rodent abundance further suggests that flea burden may influence host populations only after exceeding a critical threshold. Moderate flea prevalence may have little demographic consequence, whereas high flea infection levels could increase pathogen transmission, physiological stress, and mortality, thereby suppressing population growth. These findings indicate that the ecological responses of *R. tanezumi* are jointly shaped by climatic forcing and host–vector interactions, but that the effects are moderated by its commensal lifestyle and human-modified habitats.

### 4.3. Temporal Instability and Anthropogenic Modulation of Climate–Ecology Coupling

A key finding of this study is that the strength and phase of ENSO–rodent coupling are not stationary over time. Wavelet coherence analyses revealed a clear shift in phase relationships around the mid-1990s, followed by a marked weakening of coherence for *R. tanezumi* after 2005. This temporal instability suggests that climate–population linkages are modulated by additional anthropogenic factors. Following the SARS epidemic in 2004, China substantially strengthened rodent control programs in residential areas. Such long-term interventions likely suppressed *R. tanezumi* populations and disrupted naturally occurring ENSO-driven oscillations [34]. Such interventions may disrupt natural population cycles and decouple host dynamics from climatic forcing. The subsequent re-emergence of coherence signals after 2015 further supports the idea that climate-driven dynamics can re-establish once anthropogenic pressures are relaxed or ecological conditions shift.

These findings highlight that zoonotic systems are shaped by the interaction between natural climatic variability and human intervention, emphasizing the need to consider both factors when interpreting long-term ecological dynamics.

### 4.4. Implications for Plague Ecology and One Health Surveillance

From a One Health perspective, the observed climate–host–vector interactions have direct implications for understanding and managing plague risk. ENSO-driven increases in rodent populations can enhance the ecological conditions that support the persistence and transmission of *Yersinia pestis*, particularly in natural plague foci where wildlife hosts, vectors, and human populations intersect.

The identification of consistent 2–3-year ENSO-linked cycles and lagged responses in rodent populations provides a valuable basis for early warning systems. Because ENSO forecasts are routinely available several months before local climatic anomalies develop, SOI provides an opportunity for anticipatory surveillance. Although our study was not designed to define operational warning thresholds, persistent El Niño conditions combined with increasing rodent densities could serve as an ecological trigger for enhanced surveillance. Public health agencies could use this lag time to intensify rodent and flea monitoring, implement targeted vector and rodent control in high-risk areas, strengthen community education, and improve laboratory preparedness. Importantly, the species-specific responses observed here suggest that surveillance strategies should account for differences between wild and commensal hosts, as these groups contribute differently to zoonotic transmission pathways.

### 4.5. Study Limitations

Although this study is based on one of the longest continuous rodent surveillance datasets available from a natural plague focus, several limitations should be acknowledged. Although standardized surveillance protocols were maintained throughout the study period, potential variation in trapping efficiency and changes in field personnel over nearly five decades cannot be completely excluded. In addition, our analyses did not include other environmental and anthropogenic factors, such as vegetation dynamics (e.g., NDVI or LAI), land-use change, predator abundance, or rodent control interventions, which also influence rodent population dynamics. Future studies should integrate ENSO forecasts with remote sensing and long-term ecological monitoring to improve the mechanistic understanding and prediction of plague risk.

## 5. Conclusions

Collectively, our findings demonstrate that large-scale climate oscillations, host ecology, and human intervention jointly determine plague reservoir dynamics. Integrating climate forecasting with ecological surveillance represents a promising direction for predictive One Health management of plague under future climate change.

## Data Availability

The data that support the findings of this study are available from the authors upon reasonable request.

## Supplementary Materials

The following supporting information can be downloaded at: https://www.mdpi.com/article/10.3390/ani16152388/s1. Figure S1: Monthly (Southern Oscillation Index) SOI (A), Temperature (B) and Precipitation (C) from 1978 to 2025; Figure S2: Cross-correlation function analysis between rodent capture rates and the SOI. Panels show results for *A. chevrieri* (A) and *R. tanezumi* (B). Red boxes highlight the lag (in months) at which the strongest significant correlations were detected, indicating the lead time of SOI relative to rodent population responses; Figure S3: Best-fitting GAMs describing for flea infection in *A. chevrieri*; Figure S4: Best-fitting GAMs describing for flea infection in *R. tanezumi*; Table S1: Effective degrees of freedom (edf) and response curve characteristics of generalized additive models (GAMs) for rodent population dynamics.
