# Supplementary material for: Long-Term Evidence of ENSO-Driven Rodent Population Dynamics in a Natural Plague Focus of Southwestern China
Source: Animals (Basel). 2026 Aug 3;16(15):2388. doi: 10.3390/ani16152388 (PMC13465292; doi:10.3390/ani16152388)
Supplement: Supplementary file 1 [file animals-16-02388-s001.zip › animals-4397588-supplementary.pdf]

Supplementary Figure S1

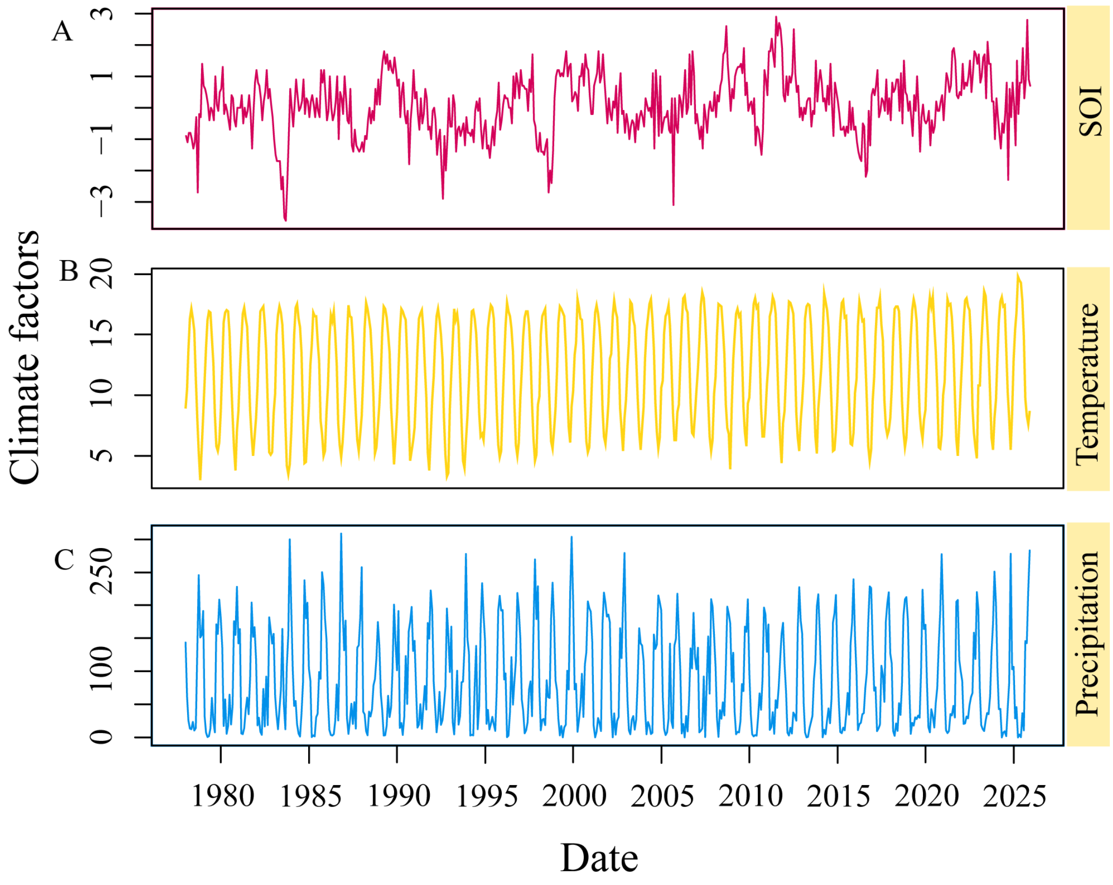

Figure S1. Monthly (Southern Oscillation Index) SOI (A), Temperature (B) and Precipitation (C) from 1978 to 2025.

Supplementary Figure S2

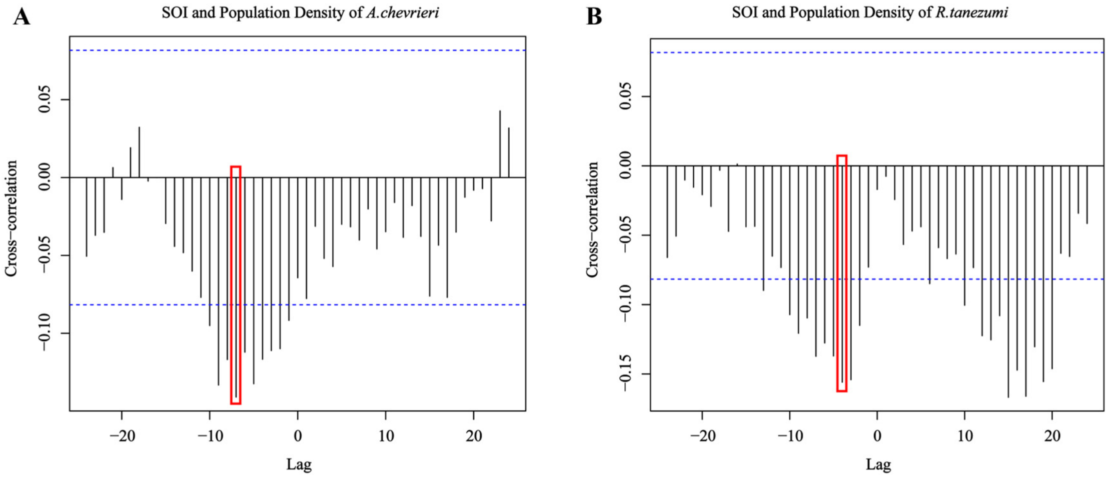

Figure S2. Cross-correlation function analysis between rodent capture rates and the SOI. Panels show results for *A. chevrieri* (A) and *R. tanezumi* (B). Red boxes highlight the lag (in months) at which the strongest significant correlations were detected, indicating the lead time of SOI relative to rodent population responses.

Supplementary Figure S3

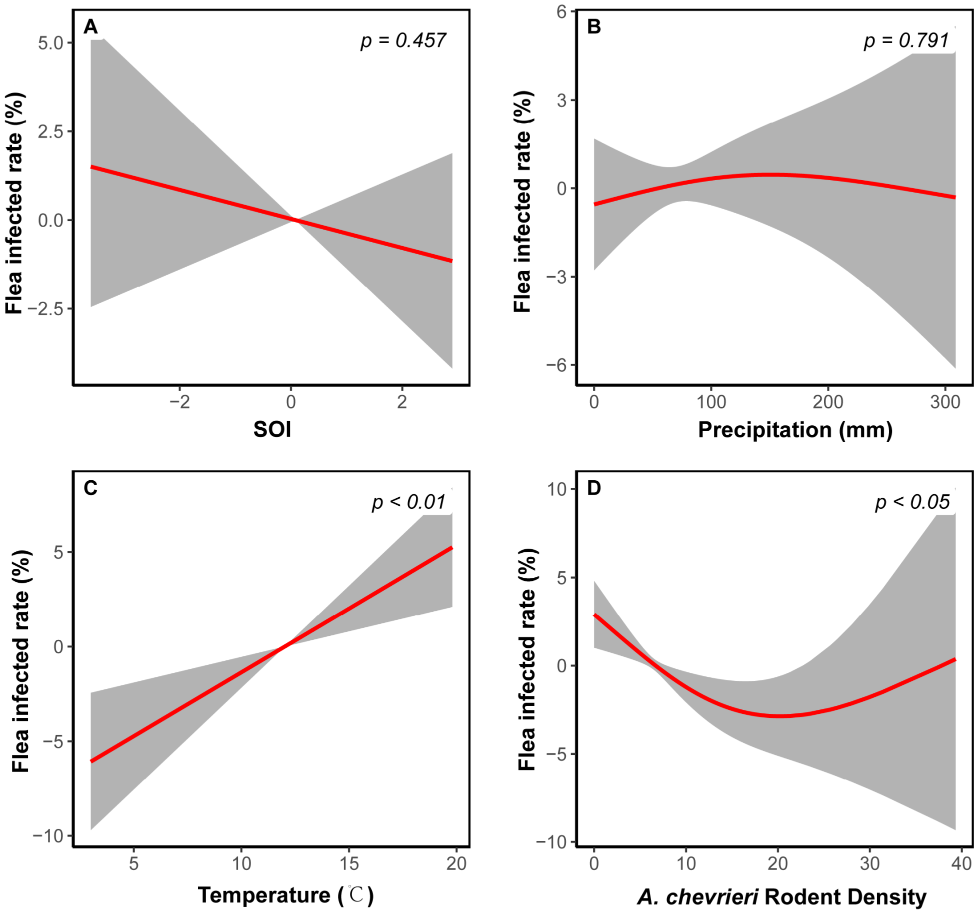

Figure S3. Best-fitting GAMs describing for flea infection in *A. chevrieri*.

Supplementary Figure S4

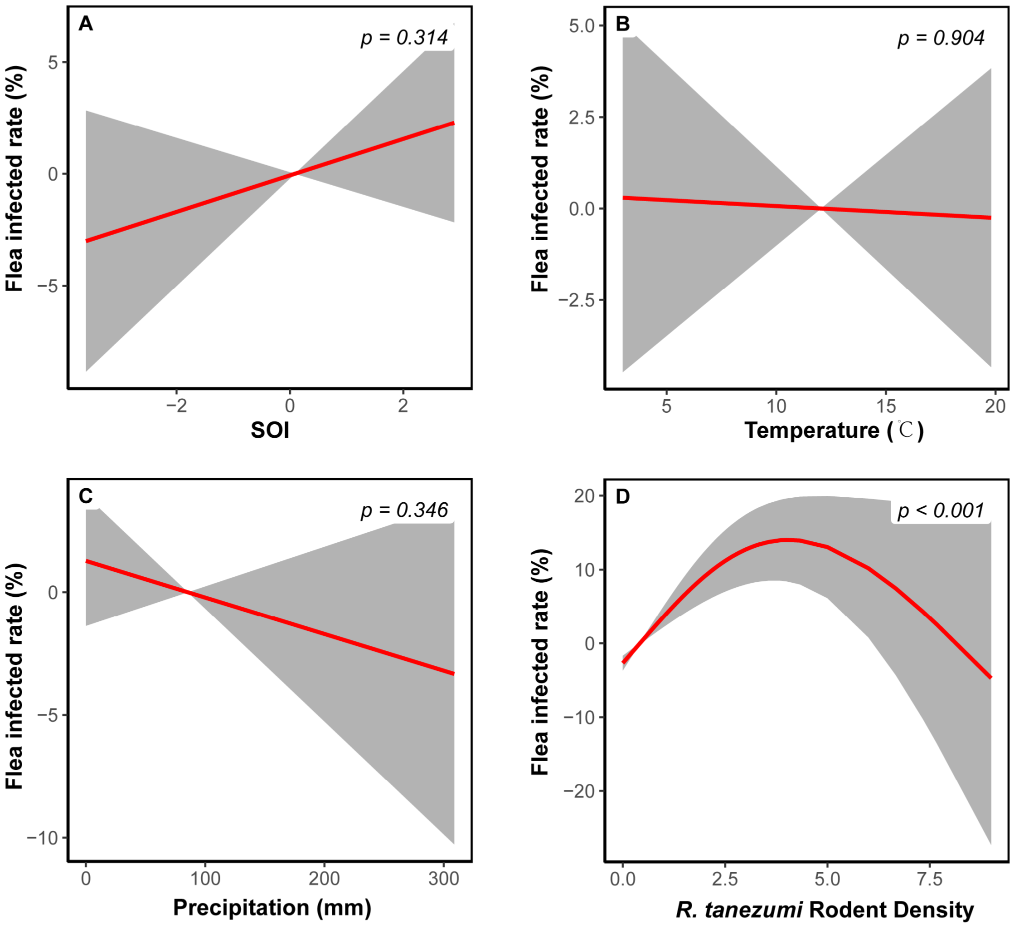

Figure S4. Best-fitting GAMs describing for flea infection in *R. tanezumi*.

Supplementary Table S1

Table S1. Effective degrees of freedom (edf) and response curve characteristics of generalized additive models (GAMs) for rodent population dynamics.

**Rodent species**	**Variations**	**effective degrees of freedom (edf)**	**Curve**
A. chevrieri	SOI	**1.631**	Non-linear
	Precipitation	**1.000**	Linear
	Flea infection	**1.406**	Non-linear
	Flea index	**1.826**	Non-linear
R. tanezumi	SOI	**1.954**	Non-linear
	Flea infection	**1.968**	Non-linear
